# Detecting Welfare in a Non-Verbal Species: Social/Cultural Biases and Difficulties in Horse Welfare Assessment

**DOI:** 10.3390/ani11082249

**Published:** 2021-07-30

**Authors:** Martine Hausberger, Clémence Lesimple, Séverine Henry

**Affiliations:** Laboratoire d’Ethologie Animale et Humaine, Université de Rennes, UMR 6552 CNRS, Université de Caen-Normandie, Station Biologique, 35380 Paimpont, France; martine.hausberger@univ-rennes1.fr (M.H.); clemence.lesimple@univ-rennes1.fr (C.L.)

**Keywords:** horses, welfare, overexposure, social norms, management practices

## Abstract

**Simple Summary:**

There is a paradox about the welfare of horses in the domestic situation: on the one hand, horses are beloved partners for most owners, but on the other hand, scientific studies are converging to show that there is a high prevalence of welfare problems. There seems to be a mismatch between theoretical knowledge and field applications. In this review, we aim at disentangling the possible factors explaining such a paradox. Among them, we consider the impact of anthropomorphic and cultural biases, popular beliefs, but also overexposure to horses with compromised welfare state, which can change owners’ representation of what is a “normal” horse, on the undervaluation of horse welfare state. We suggest that, rather than simply having knowledge on what should be done, identifying the horse welfare state using validated animal-based indicators is essential to identify and promote best practices.

**Abstract:**

Horses were domesticated for more than 5000 years and have been one of the most emblematic species living alongside humans. This long-shared history would suggest that horses are well known and well understood, but scientific data raise many concerns about the welfare state of most domestic horses suggesting that many aspects have been largely misunderstood. In the present review, we will examine some of the possible human factors that may explain the huge prevalence of welfare problems, despite horses being of special importance to humans. First of all, as horses are non-verbal, current management practices rely upon what one thinks is good for them, which opens the way to subjective interpretations and projections, based on one’s own subjective experience but probably still more on cultural/social norms and influences, traditions and beliefs. The lack of recognition, identification, or even the misinterpretation of signals are other potential reasons for welfare issues. Lastly, the over-exposure to animals with expressions of compromised welfare may lead to lower sensitivity of owners/professionals. That is why we lastly suggest that instead of simply providing information on what to do, we should promote validated visible indicators that leave less room for personal interpretation.

## 1. Introduction

There is a fundamental paradox about domestic horse welfare: while all horse owners and caretakers, whether professional or private, wish the best for their horses [1], data issued from different studies raise many concerns about the welfare state of most adult horses maintained under domestic situation [2]. For instance, a recent survey on 1850 horses indicates that 82% of horses sampled display behavioral problems in the stable context [3]. 

Horses have been domesticated for more than 5000 years and have been one of the most emblematic species living alongside humans [4]. Their roles in human society have varied over time and, as a result, people’s perception of horses and the importance attached to their well-being have evolved along the same line. However, never before, since their rise for sport and recreation, after the emergence of mechanization in the 1950s, have horses been so unanimously regarded as “friends” or “companions”, thus as individuals deserving care and a good quality of life [4,5]. This long shared history would suggest that horses are well known and well understood, but the paradoxical state of their welfare reveals that many aspects may have been actually misunderstood. One of the reasons may be the mixed status of horses, which do not live in the house like dogs but are much more of a companion than animals raised for production [4]. However, other reasons, more related to human functioning, may also be evoked. Thus, Visser and Van Wijk-Janssen [6], using an online survey, found, for most of the 4267 respondents (“horse enthusiasts”), a discrepancy between their knowledge of the basic needs of horses and the way they kept their own horses. For example, a high proportion of them cited 24 h-stabling as a major factor accounting for a reduced level of welfare, mentioning that horses are adapted to move around all day long, but most of them had actually their own horses housed permanently or semi-permanently in stalls. According to the authors, there was a gap between “conceptual” (i.e., knowing that) and “procedural” (i.e., knowing how) knowledge that prevented these respondents from applying appropriate management procedures. They identified four categories of respondents with different response patterns: one category composed of only 2.7% of the respondents had theoretical knowledge and applied it to their horses’ management, while in the remaining three categories, conceptual knowledge was either absent, partial or there but not applied. For one category, represented mostly by male owners, obviously involved in sport competition, performance was “the” indicator that horses were well. Of course, since such surveys are on the voluntary basis, it is most likely that a good part of the equestrian population, potentially less aware or concerned by horse welfare issues, had not been included. Remarkably though, similar discrepancies are found for a variety of domestic animals and situations: for instance, Chilean farmers mention that the use of knife for castration of lambs is more painful than rubber rings, but still use a knife [7]. 

There are many reasons why people may not actively promote their own horse’s welfare through appropriate management: they may not perceive that there is a problem; they may misinterpret signals; they may follow erroneous advice; and/or they may be influenced by culture, social networks and media. Thus, information in magazines and on the internet is now having an important impact on people’s thinking about animal welfare. A study, based on articles published in a Finnish equestrian magazine, found statements such as “a horse that works has no welfare problems”, “competition horses are kept well in order to compete”, or “cleanliness is essential in equine stable” [8]. Stereotypes and biases are frequent in the equestrian community: in Fenner et al.’s study [9], mares are described by respondents as “bossy and bad” and stallions as “highly trainable but difficult”, which of course does not rely upon any scientific evidence of clear sex differences in temperament or training abilities. Birke et al.’s study [10], on the basis of self-administered questionnaires filled by 1630 persons, reveals how much peer pressure and consensus is shaping how people construct ideas about animal welfare. There is a “yard culture”, the place where different owners have their horses kept, where social processes of inclusion and exclusion influence individual decision making. Breaking with the local management routines, even for the horse’s sake, puts the owner at risk of being socially excluded from the community. 

In the present literature review, we will examine further some of the possible human factors that may explain the huge prevalence of welfare problems observed in scientific studies in horses, despite horses being of special importance to humans.

## 2. Different Sensory Worlds and Adaptations: From Perception to Empathy

Horses and humans live in different “Umwelt” (meaning “subjective universe”), a term developed by von Uexkull [11] to characterize the particular perceptual world of a species or even an individual [12]. Thus, the eyes of horses are very large (more than seven times the size of a human eye), laterally placed (on each side of the head), providing the horse with 180° panoramic vision in the vertical dimension and 140° monocular vision on each side [13]. Horses have more rods than cones and thus perceive, better than humans, objects in relative darkness and are very sensitive to movement perception, which may explain both horse reactivity to moving objects and their ability to detect subtle movements (e.g., Clever Hans), but their color vision is less rich than that of humans [14]. Horse audiograms (curves based on responses in discrimination studies at different frequencies and intensities) reveal that they perceive a broader range of sound frequencies (5 Hz–33 kHz vs. 20 Hz–17 kHz in humans), including ultrasounds [15]. Behavioral experiments suggest that horses may use olfactory cues to discriminate sexes from urine [16], familiarity from feces [17], conspecific stress from blood [18] cues. They may associate the visual representation of a familiar human to its odor [19]. Horses are also able to detect a fly sitting on their skin, as shown by skin tremor. Studies using calibrated filaments (von Frey thin filaments) applied at different parts of the body show that horses can perceive a filament of 0.02 g/m density, (or even 0.008 g) which reveals a much higher tactile sensitivity than humans [20] (Gueguen et al. in prep). Horses therefore may be sensitive to environmental cues that we are not aware of. Tactile sensitivity is certainly largely under-evaluated as riders’ actions through legs and hands (conducting to the bit and thus the mouth) may be perceived by horses as quite rough considering these thresholds. Horses may express compromised welfare through lowered visual, auditory and tactile reactivity as shown in apathetic horses [21,22,23], which may be misinterpreted as being voluntary acceptance of human contact and actions. This apathy can result from learned helplessness, i.e., learning that movements to escape pain or discomfort are just useless. Sudden reactions to sounds that humans do not perceive (e.g., ultrasound whistles for hunting dogs) may be punished as being just disobedience or undesirable aimless behaviors. Many other such examples of misunderstanding due to this discrepancy between these species-specific Umwelt could be given in the daily interactions between humans and horses. 

Similar difficulties arise when considering respective behavioral and physiological adaptations, be it the food regimen, daily rhythms or modalities of pain expression. Humans, as an omnivorous species with a diet based on time-limited meals and relatively high energy/protein intake, have difficulties to represent the digestive physiology of horses, adapted to low energy intake and semi-continuous feeding [24]. It is difficult to imagine that resting behavior is not limited to night time and that horses may thus be disturbed by human noises and movements in the stable at day time while on the contrary they may feel the need to feed or move at times of the night. Pain/sickness expression, which may include inhibitory reactions, can be very subtle for a human eye and thus unseen [25]. 

Difficulties in representing the other’s perceptual world and needs and interpreting the other’s signals and expressions are not proper to human-animal interactions; they are common to all situations where adult verbal humans are confronted to non-verbal individuals. The challenge is the same when caregivers are confronted with non-verbal humans, whether non-speaking persons with autistic spectrum disorders [26,27] or even newborn human babies [28,29], who live in a very different sensory world and have very different needs from those of human adults. 

These difficulties to comprehend the world and needs of others lay the foundations of misunderstanding and constitute a real challenge for those in charge of the management and good welfare of these non-verbal individuals. Language use has probably contributed to our lower abilities to detect other types of signals. This means that current management practices rely upon what one thinks is good for the individual concerned, opening the way to subjective interpretations and projections, based on one’s own subjective experience but probably still more on cultural/social norms and influences, traditions and beliefs. It has been proposed that better human attitudes towards animals could be based on anthropomorphism and empathy [30].

Anthropomorphism is considered a cognitive bias in that it involves beliefs about the extent to which animals may have, like humans, awareness, thoughts and feelings [30]. While it may be useful at some point in understanding the parallels between animal and human suffering, anthropomorphism can pose a real risk when it leads to treating animals as if they were mentally or physically the same as humans [31]. There are some associations between anthropomorphic beliefs and empathy towards animals, but these concepts correspond to two very different constructs [30].

Empathy, defined as “affective response that stems from the apprehension or comprehension of another’s emotional state or condition and is similar to what the other person is feeling or would be expected to feel” [32], is based on taking the other’s perspective. It is seen as a way of apprehending how to promote good welfare. “Empathic” vets and farmers identify pain better [33], and empathy towards animals is more present in activists of animal rights [34]. However, the question really is to which extent empathy may be an “honest” perspective taking. Attributing the right emotion to another individual does not mean that one knows what induced this emotion. This may explain the difficulty of going from a “conceptual” knowledge to a “procedural” knowledge: one can have learned what is appropriate but not comprehend the effect on the target individual, because of the lack of similar experience. This is the whole problem of welfare assessment through resource-based indicators: one can learn that a horse must have access to feeding all day long, but it does not necessarily inform us about how the horse really feels about having an empty stomach, just because this is an unshared experience. This is why positive horse owner attitudes, instead of resulting in positive welfare, may sometimes in fact result in negative welfare [35].

Animal-based indicators are more likely to be useful candidates for developing empathy, but most training strategies are not based on learning to identify them and make parallel with human emotions and feelings. Thus, “identification” was a key aspect of cats’ emotion detection by humans in Thibault et al.’s experience [36]: accuracy of recognition of cats’ emotions was not improved by experience or liking cats but was better when subjects “identified” to cats (i.e., considered “they are like us”). For the authors, identification may have induced “decoders” to attempt to take the cat’s perspective and hence to be more motivated to engage in cognitively more demanding decoding strategies. This could be a key aspect leading to take active measures to prevent poor welfare. For Hills [34], identification goes beyond empathy by including positive and negative aspects and therefore has more chances to give a better view of the actual animal’s state. There are indeed parallels between humans’ and animals’ expressions of compromised welfare: thus, depressive individuals are withdrawn, avoid sensory stimulation and have a characteristic posture [37]; back problems induce stiffness and bad mood/aggressiveness [38,39]; obesity is obviously beyond species-specific boundaries. However, there are many obstacles to proper identification of poor/good welfare state and to the resulting appropriate management changes.

## 3. Anthropomorphic Biases

### 3.1. Stall = Security

**Figure 1:**
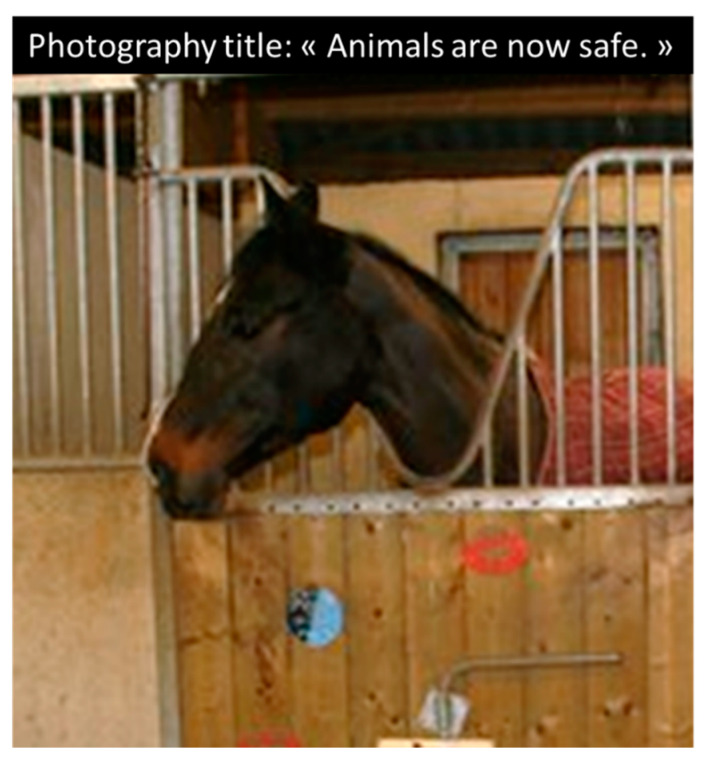
Example of popular fact reported in the French press (Sud-Ouest published on the 4 February 2020): it is said that a group of horses living out in pasture have been fortunately saved and now placed in “security” in stalls. Photo credit: Martine Hausberger. See also https://www.sudouest.fr/2020/02/04/gard-un-proprietaire-maltraite-ses-chevaux-ils-lui-sont-enleves-et-confies-a-une-association-7151867-6095.php?nic (accessed on 4 February 2020).

Despite the growing knowledge within the equestrian community that horses need free movement and companionship, most horses are still living permanently in individual stalls [40,41]. Beyond the obvious practical reasons for such a practice (e.g., having a clean horse available for work at all times), owners give an array of “good reasons” for keeping it, such as weather protection, limitation of the risks of injury or of the consumption of toxic plants [42]. In addition, when a horse rarely goes to paddocks and/or finds itself there without food or companion on a bare soil, it may stay at the entrance, and this is unfortunately often interpreted as a horse feeling better in its stall. Because this is such a common housing system in the professional horse community, its deleterious effects are too rarely mentioned to challenge the system. In the study by Birke et al. [10], some horse owners who had their horses in yards reported that they had switched foryards where their horses were allowed to graze instead of individual stalls in the former yard, but they were then considered as being “fussy”. On the contrary, problems with pasture housing, including fences’ quality, appeared as the first source of worries for professional stakeholders in Horseman’s study [42]. The fact is that there are more worries from the animal rights organizations about horses being supposedly “abandoned” in pastures than about horses in “security” in their stalls (Figure 1). Lastly, in Visser and Van Wijk-Janssen’s study [6], one cluster of female “horse enthusiasts”, who considered their horse as a “friend” or a “child”, mentioned they knew that horses needed free movement but were those whose horses were in the most restricted spatial conditions. Protection and control over the security of “dependent” partners may lead to inappropriate overprotection measures. The other bias is that measures such as “environmental enrichment” or broadening the visual horizon may be viewed as sufficient compensations.

### 3.2. Visual Horizons and Enrichment: Where Horses and Human Views May Diverge

Humans enjoy having windows offering views on the outside world; therefore, it is quite logical for human owners to provide outdoor windows to their loved pets and other companion animals. However, perception of landscapes may lead to quite opposite reactions in animals kept in restrictive spatial conditions: real view or broadcast of landscape’ pictures led to increased frequencies of motor stereotypies in captive European starlings housed in single cages [43,44]. In horses, studies performed by Cooper et al. [45] have shed new light on the impact of outdoor windows on horses living in single stalls, showing that they were associated with an increase of vigilance/alert postures. The presence of outdoor windows appeared amongst the primary factors predicting stereotypic behaviors in an epidemiological observational study conducted by Lesimple et al. [40] on more than 300 horses. More recently, in a study performed on 32 sport horses kept in permanent single stalls in the same facility, Lesimple et al. [46] showed that the horses that had a visual access to the outside, but no close social contact expressed more “excitation” and more visible stereotypic (weaving) behaviors than horses living in an indoor stable but with close social contacts which expressed more relaxed behaviors. In a joint study on 42 Arabian broodmares, it appeared that the same mares behaved quite differently when they spent time in stalls with an outdoor opening than when there was a grid at the door’s top: they performed less stereotypic behaviors and spent more time foraging and resting when they had grids on the half door than those who could have the head outside and watch out-of-reach conspecifics and humans walking around. It has been suggested that stereotypic behaviors, and especially, weaving, may reflect frustration of restricted movement and access to congeners [45,47], which reminds of caged mice which repeatedly gnaw at their cage bars, as frustrated attempts to escape [48]. Possible horse frustration as a result of visual but not direct contact with conspecifics has also been mentioned by Redbo et al. [49] and Hockenhull and Creighton [50]. 

**Figure 2:**
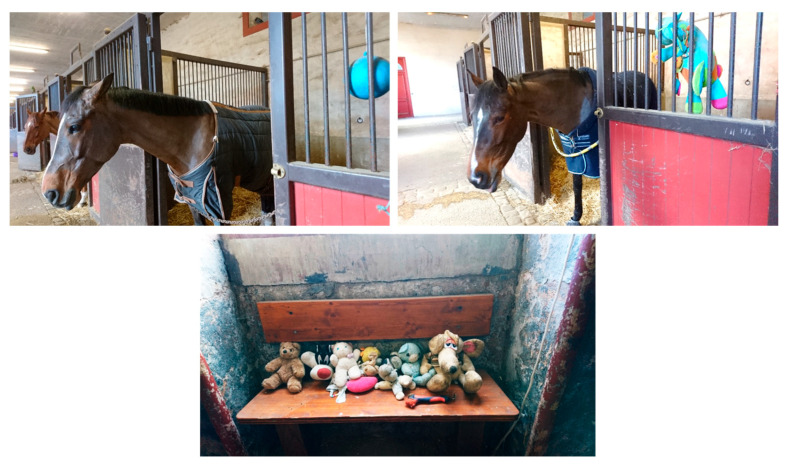
Examples of environmental enrichments placed in the stall and as a stock for turnover. Photo credit: Martine Hausberger.

The observed discrepancy between conceptual knowledge (“horses need free movement”) and actual choices of some horse owners to keep their horses in single stalls [6] must induce conscious or unconscious uneasiness when it comes to horse welfare. Coming from attempts to compensate the impoverished management conditions offered to zoo animals [51], the concept of “enrichment” has spread to the world of companion, laboratory and even farm animals. This remains however a poorly defined concept that has opened the way to a large commercial market where the “enrichments” proposed include a whole variety of “natural” and manufactured objects. Of course, this sounds as an easy way to promote the horse’s well-being without the constraints related to offering more appropriate life conditions, and many horse owners have added such enrichments in the stalls, as ways of fighting boredom and frustration. It is however much less clear from the horse’s point of view. Jørgensen et al. [52] have shown that amongst an array of different enrichment items, only those related to food (e.g., hay, soil, foodball) attracted the attention of horses for a significant amount of time. Lansade et al. [53] found that young horses living in “enriched” conditions had a better welfare and relationship to humans that those kept in conventional (i.e., impoverished) conditions. However, in this study, enrichment consisted in an array of aspects, such as being turned out with conspecifics, having hay ad libitum as well as music or other more artificial types of items. It is therefore difficult to assess whether it was “enrichment” (i.e., etymologically “more than rich”) or the fact that more appropriate feeding and housing conditions were offered that led to these results. To our knowledge, there is no evidence at that stage that the numerous objects found nowadays in stables (e.g., balls, lick-it, plush animals, baby teeth rings, etc.) have a positive impact on horse welfare (Figure 2). Mason et al. [54] have shown that minks living in single cages in breeding farms would work hard for an access to water and food but that access to toys offered as enrichment was their very last choice. The more and more frequent observation of plush animals in stables, and especially plush horses and unicorns, illustrates how humans make projections on how horses see the world, imagining that plush animals or other toys will trigger their interest and imagination, which is what plush animals do for children [55]. It would not be a problem per se (as horses can just neglect the object) if it were not a means of diverting the owner’s attention and consciousness from the real welfare problems. With an “enriched environment”, one can feel that horse welfare is ensured. It is also interesting that in some studies, providing hay, straw bedding or contact with a conspecific is described as providing enrichment [56,57], whereas it is just providing appropriate care instead of the impoverished conventional conditions. It would thus be worth considering replacing the term “enrichment” by “compensation of conditions” when providing items that are thought to help and by “appropriate” conditions when it is just coming back to what horses are adapted for. 

Other aspects, such as the belief that all horses like tactile contact (like us humans) or that cleanliness of stables is a major aspect for horse welfare, reflect also our propensity to project our own needs, preferences and habits on companion animals [58], considering that tactile contact can be perceived as quite invasive [59,60] and the fact that first thing a horse does when coming out of stall is rolling in the mud. These are thus further illustrations of anthropomorphic biases.

## 4. Cultural Representations and Social Norms

### 4.1. Obesity

It may not be mere chance if the prevalence of obesity has kept increasing both in humans [61,62] and domestic animals in the last decades. For instance, it has been estimated that 22 to 41% of adult dogs and 45% of cats were overweight [63,64,65]. Although not clearly stated to our knowledge, the onset of this increase may well lie on the post second world war, after years of deprivation for both humans and domestic animals [66]. Food was scarce, with little variety, and especially “rich food”, like meat, butter and white bread was missing and mostly sold through the black market. The increase in food consumption and the consumption of food as a comfort factor through increased protein and sugar levels have spread to the detriment of food quality. Human health problems are related to the over consumption of meat, rich milk products and compulsory eating [67,68]. 

All epidemiological studies, whatever the type of body condition scoring, converge to show a similar high prevalence of overweight/obese horses in different western countries [69], e.g., 45% out of 319 riding horses in Scotland, 24% in Icelandic horses, 32% in Italy and Germany [70,71,72]. Although often attributed to the “leisure horse community”, opportunistic recordings have shown that this is a common problem in riding schools or even sport horses as well [40,73,74]. Different studies have shown that horse owners/caretakers underestimate their overweight horses’ body condition [66,75]. Owners underestimated more than they overestimated their horse’s body condition as compared to measures performed by an experienced experimenter in Jensen et al.’s study [71] and agreement was poor (Kappa = 0.21). It is worth noting that in studies in western countries, reports of underweight horses are rather anecdotal. Fureix [73] measured the body condition of 59 adult horses from three riding schools using the 5-point body condition scoring of Arnaud et al. [76] and found that 15 of them (25%) were overweight. In parallel, the caretakers were invited to fill a questionnaire on the chronic health and estimated body condition for each horse. It appeared that all these horses were considered as having an “optimal” body condition by their caretakers. Since excess body weight is a source of health problems, this is becoming a major issue [69].

It results from historical background and cultural western influences that “caring for others” is feeding them well. No wonder then that this typical human problem extends to those people love and care for, which are pets and semi-companion animals like horses. Even in the competition community, judges may rate higher horses with larger condition [77] influencing further representations of “normality”. 

### 4.2. The Weight of “Traditions“: The Example of Bit Use 

**Figure 3:**
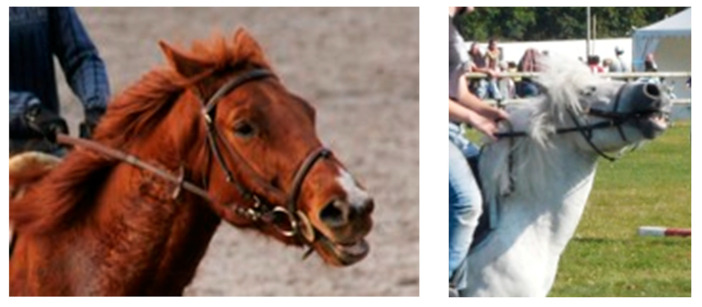
High hands and tensed reins are associated with high neck and undesirable behaviors in horses, such as mouth opening. Photo credit: Martine Hausberger.

Bits have been used to manage horses since ancient times [78], although representations of riding Etrusceans seem to indicate that other techniques, such as body indications, may have prevailed at some stages. Since the pioneering work of Cook [79,80], indicating that bit use was associated with potential head, jaw and neck discomfort or pain, “bitless bridles” have been sold all over the world, mostly, but not only, to leisure riders. Nevertheless, bitless riding is still practiced by a rather small part of the equestrian community, and the concept leads to conflicting positions. It is obvious that the bit, placed in a very sensitive part of the horse’s body, may increase the effects of harsh or unexperienced actions of riders’ hands: Lesimple et al. [39,40,81] showed that hand positions and rein lengths were determinants not only for bitted horses’ reactions and neck positions during riding lessons for beginners but for predicting risks of chronic back problems. First reactions of horses to escape pain in the mouth will mostly be to raise the head, open the mouth and therefore elevate their neck [82] (Figure 3). In more advanced riding, strong actions via the bit, such as rollkür or some techniques of advanced dressage, induce mouth pain that leads to escape behaviors, such as tongue prone/play, opening of the mouth, head tossing/nodding [83,84]. It has been suggested that some of the oral stereotypies observed in the stall in some sport horses may correspond to a chronic version of escape behaviors performed during repeated training sessions [85,86]. 

One can therefore wonder why horses are commonly bitted, especially in the professional horse industry. This is especially true for beginners or persons with disabilities who cannot master their hand (and body) actions [87]. It is even common to see horses being bitted just for being led in hand and walked with a young rider on top. This tradition of bit use may be related to the fact that almost all representations of ridden horses are with bitted bridles, to the likely point that even children may feel they are not really riding if they do not have a “proper” bridle. If explained that actions on the bit may be painful to the horse, they may ask their teacher and then be responded that “bits have always been used, if they were a problem, their use would have stopped” (MH, pers. obs.) In most cases, persons in charge have not particularly thought about why they put bitted bridles on the horses. If asked, they will cognitively answer that this is for security, even saying though that bits do not stop a horse from running away if it wishes so (MH, pers. obs.) It is also well known that using stronger bits has never solved management problems of ridden horses [80]. Beliefs, based on a long cultural influence, therefore constitute an obstacle to taking useful measures to prevent horses and ponies used for young beginning riders to suffer from their unexperienced riders’ actions. This is of course a vicious circle as pain and discomfort lead horses to express undesirable behaviors and threaten young riders’ security, but as illustrated by the rollkür debate, problems related to bit use are not restricted to unexperienced hands. Riders may want to stop undesirable behaviors, such as mouth opening, resulting from harsh bit actions by using tight nosebands that further increase the discomfort [88,89]. Alternative dressage techniques have been proposed to preserve the horse’s mouth [90] suggesting that, in skilled hands, its more the way the bit is used than its presence itself that may generate pain and discomfort. Actually, the impact of the bit on the mouth would be mostly related to rein tension according to Manfredi et al.’s study [91]. Inappropriate hand actions with bitless bridles, by blocking neck movement, may also induce discomfort during sessions (Lerch et al. in prep.) However, it is highly likely that the bit acts as a magnifier of this discomfort. 

This example illustrates how routine procedures are rarely questioned nor is questioned why it is so and where it comes from. Interestingly, in Horseman ‘s study [42], stakeholders mentioned that riding/training were factors affecting horse welfare, mostly through inappropriate gear and use of aids. The study does not mention what exactly was meant behind these statements, but behavioral problems related to riding and gear appeared clearly in Hockenhull and Creighton’s study [92]. Whereas riding appeared in the fourth place as a source of welfare problems, Lesimple et al.’s study [40] showed that inappropriate hand positions were the second predicting factor for stereotypic behaviors performed in stall. 

### 4.3. Community Beliefs: Positive Reinforcement

As very well demonstrated by Warren-Smith and Mc Greevy [93], on the basis of questionnaires filled by 830 professional horse trainers in Australia, the use of negative reinforcement is largely considered as the only efficient way of training horses, and when, more rarely, positive reinforcement is mentioned, it is mostly restricted to tactile reinforcement such as patting the neck (see also [6]). This is a unique particularity of the equestrian community as positive reinforcement has always been predominant in training wild captive animals for shows in circuses or medical training in zoos [94] and is increasingly recommended for laboratory animals and dog training, especially service dogs [95,96,97]. 

Although the use of positive reinforcement may be more difficult to implement during riding sessions, this does not explain why it is so scarcely used in the horse trainer community. There is a belief that it would increase biting during and outside training sessions, which no scientific study has confirmed [98,99]. Another aspect is that horse owners want horses to work out of love for themselves and not because of food, a statement sometimes reinforced by the fact that positive reinforcement is banned from most methods of “natural horsemanship”. This is of course opposite to the findings that food reinforcement during training promotes human–horse relationship and that tactile contact is not reinforcing enough to promote learning and bonding [100,101]. The opposite position, trying to please the horse by offering food item by hand any time outside training sessions, may lead to quite opposite effects such as constant expectations that can lead to biting and other undesirable behaviors.

## 5. “What the Eye Doesn’t See”

Major chronic problems are overlooked as horses have very “discrete” or “undirect” ways of expressing chronic pain [25].

Digestive problems are one of them: different studies have evaluated the prevalence of gastric ulceration to 30% to 93% in working horses [102,103,104,105,106,107]. Because there are no clear clinical signs described yet, this remains an unsolved issue and no major changes in feeding management are provided, leading to lasting suffering, development of unexplained abnormal behaviors or excess vacuum chewing to promote saliva production and alleviate the constant pain [108,109].

Back problems constitute “one of the most common and less treated problems in the horse” [110] and another common issue with the human health. Back disorders are recognized as a common problem in working horses (review in [39]). The estimated prevalence varies from 35% [111] to 100% [112] of the ridden horse population. There are no sex nor age effects, but there are strong differences according to the type of work practiced, with the highest prevalence found in riding school horses, followed by racehorses, and the lowest in leisure horses [113,114], (review in [39]). Fonseca et al. [115] showed that the location and prevalence of back disorders varied according to the discipline in western riding. Although there is an array of behavioral signs of back pain, both at work (e.g., bucking, reluctance to turn) or outside work (e.g., aggressiveness, avoidance of riding gear) [116,117], these signs remain unspecific and may be misinterpreted as reflecting bad temperament or bad mood [38,39]. Physical manifestations, such as stiffness, restricted gaits and postural changes may not be visible for non-specialists while lameness, which sometimes results from back problems, may be overlooked or attributed to peripherical aspects [118]. More recent studies have shown that body morphometry and especially neck shape could reflect chronic muscular tensions along the spine [119] and welfare state [120]. The fact that they differ more according to the type of work than the breed confirms the idea that riding techniques play a major role in preserving or compromising horses’ back health [39,79,121]. Awareness of horses’ potential chronic back pain would thus be essential. However, Mc Greevy et al. [122] showed that only 11 out of 236 ponies with sore backs were identified as having back problems by their owner. Confirmation of the under-evaluation of this problem by stakeholders has appeared in Lesimple et al.’s [121] study performed on 161 horses working in 17 French riding schools. Assessment of back health was performed by manual palpation by an experienced therapist and/or sEMG measurements (a classical tool for assessing chronic back pain in humans). In parallel, a questionnaire on potential chronic health problems, including back problems, was given hand to hand to the caretakers. Physical measures indicated that about 40% of the horses were affected by back problems, whereas this number was only 12% when considering the caretakers’ reports. Moreover, there were large discrepancies also in which horses were affected and thus agreement between both types of evaluations was quite low (Kappa = 0.08, 95% CI: 0–0.32). Strong differences appeared between the schools (0 to 85%) in the prevalence of horses affected. Interestingly, in a few schools, the caretaker over-evaluated back disorders. On the contrary, in places where most horses presented back disorders, caretakers were confident that their horses did not have back pain. Owners or caretakers may have personal interpretations of behaviors they assume to reflect discomfort or pain [123]. It is also possible that animals with pain have become the norm (see part on overexposure). Thus, one could think that in schools where the caretakers reported more back pain problems, people were more “sensitive” in a general way to their horses, thus promoting less constraining riding techniques as well as more positive environmental conditions. 

## 6. Overexposure

### 6.1. The Example of Stereotypic Behaviors

Stereotypic behaviors are fairly well-known in the equestrian community, being often qualified as vices. They are most generally considered as undesirable behaviors either because they reflect a welfare problem in the stable and/or because they are supposed to compromise the horse’s health (e.g., dental problems related to cribbing) or lower the horse’s market price [124,125,126]. Nevertheless, they constitute one of the commonest expressions of compromised welfare in all countries [40,127,128] showing that the roots of the problem have not been dealt with. 

**Figure 4:**
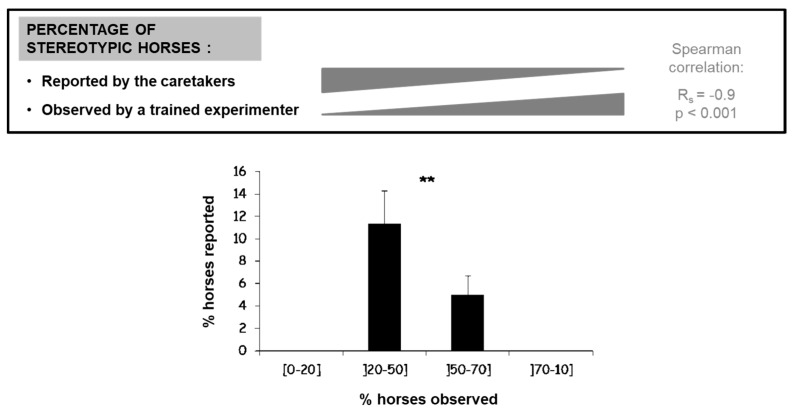
Discrepancy between observed (i.e., direct observation by trained experimenter) and reported (i.e., questionnaires to caretakers) prevalence of stereotypic horses per facility (N = 26 riding schools, 360 horses). Note that there is no report of stereotypic horses in the facilities where most or all horses were observed performing stereotypic behaviors (Spearman correlation). **: Kruskal Wallis test, *p* = 0.02, Adapted from Lesimple et Hausberger [129].

One possibility is that their prevalence in a facility is underestimated to the point that it does not look as reflecting enough welfare problems to elicit management changes. In order to look at the evaluation of stereotypic behaviors by caretakers, Lesimple and Hausberger [129] compared the observed prevalence (based on 18 h of ethological observation per horse) of stereotypic behaviors in 373 riding school horses of varied sex, age and breed with that reported by the person who knew the best the horses (riding school’s owner or caretaker) in each riding school (N = 26; 14.3 ± 1.5 horses per school). Management was “conventional”: individual stalls, meals of pellets and hay used in riding school lessons from beginners to advanced level. The respondents were asked to answer a questionnaire for each horse about the presence of chronic disorders (e.g., allergies, colics, etc.), including the presence and type of stereotypic behaviors (e.g., head nodding or tossing, weaving, box walking, cribbing/air sucking) but also abnormal repetitive behaviors (e.g., compulsive licking or biting of environment, compulsive head movements, “vacuum” threats, keeping open mouth, rubbing teeth on the door, teeth or lips chattering, “tongue play”). Questionnaires were given hand to hand, and all professionals said they knew about these behaviors, although some “major” forms (cribbing, weaving, head tossing/nodding) were better described than others. The results showed a high discrepancy between the prevalence found by direct observation and that reported by the respondents (Figure 4): direct observations indicated that 37% of the horses presented at least one type of stereotypic/abnormal behavior whereas the questionnaire reports indicated a prevalence of 5%. Moreover, there were reports of stereotypic behaviors in only 13 schools, whereas direct observations found 24 riding schools involved to varying degrees. As expected, agreement was therefore poor between both evaluations (Cohen’s kappa = 0.14). 

Different non-exclusive hypotheses could explain these differences: reluctance of persons in charge to recognize the presence of behavior/welfare problems, horses stopping behaviors in presence of caretakers (after punishment for performing them), lack of recognition of some abnormal behaviors. Indeed, non “classical” stereotypic/abnormal behaviors were still less reported by caretakers, but not to the point that they could explain the huge discrepancy between observed and reported prevalence: even weaving, a very visible and known stereotypic behavior, was largely underestimated. Further investigation shed a new light on these results: it appeared that, when considering the riding school scale, there was a strong negative correlation between the degree of discrepancy between both evaluations and caretakers’ reported numbers. Thus, the facilities where caretakers indicated an absence or very low prevalence of these behaviors were either those where there were none or few stereotypic horses or those where direct observation indicated the highest prevalence of these behaviors (e.g., the caretaker of a facility where 100% of horses showed some form of stereotypic behavior indicated that there were none). 

The authors concluded that there were two major issues in detecting these behaviors: potential lack of identification and overexposure. Thibault et al. [36] argue that “identification” is a primary factor in decoding others’ emotions, by enhancing the motivation to put cognitive effort in the process. Interestingly, in this study, the only three schools where professionals expressed a strong concern about their horses’ welfare had, for 2 of them, a low prevalence of stereotypic behaviors (0 to 10%) and a corresponding low observed report (0%) whereas the third had a higher prevalence (30%), but the report was quite accurate (30%). As suggested by Thibault et al. [36], more “identified” individuals may put more efforts in detecting signs of poor welfare (and try to prevent it to occur). One major problem is the lack of mention of the “non-major” abnormal repetitive behaviors (all head, tongue and other repetitive behaviors, other than cribbing, crib-biting, weaving, pacing) in medias but also more regrettably still in the scientific literature, whereas they constitute a large part of the expressions of poor welfare [40,47,84]. The main point of interest in this study though was the “overexposure effect”. Overexposure leads healthcare staff to under-evaluate pain levels in hospital services [130,131]: when a majority of subjects exhibit the same expressions of discomfort or pain, these expressions become the norm for the caregiver. This is in the same line as a previous study indicating that owners of stereotypic horses had a lower evaluation of the potential negative impact of these behaviors than owners of non-stereotypic horses [132]. 

Discrepancies can be observed in the literature concerning their prevalence in adult working horses (from 1 to 96%, e.g., [84,133]). A thorough examination of these studies reveals that this may be related to the types of method used: surveys (questionnaires to caretakers) and direct observations by researchers led to different results, with prevalence varying from 1 to 10% in the first case and from 22 to 97% in the second.

### 6.2. Other Examples of Overexposure

As mentioned earlier, there is such a high prevalence of overweight horses in the western societies that it is described by owners as a social norm [134] and that many horse persons, including professional stakeholders, nowadays consider horses with objective optimal body scores as being abnormally thin. This change in representation is a major problem as it means that no measure is undertaken to work on the roots of the problems of obesity but also that horse owners and caretakers that have a good management of their horses’ feeding are at risk of being accused of malnutrition. 

Another example concerns horse body conformation as a result of inappropriate riding techniques. As mentioned earlier, a vast majority of riding school horses (but not only) has back problems that are reflected in their overall back shape and especially in a flat/concave neck shape and posture that becomes chronic, that is, it can be observed outside riding sessions: instead of presenting a normal curved neck/back line, the neck/back line remains flat or hollow, resulting in stiff movements and reduced strides that are quite characteristic of back problems [111,119]. Whereas these characteristics are easily detected by competition riders once their attention has been drawn to them, riding school staff are almost unable to even conceive that a neck shape can be different from that seen in their own stable daily (MH, pers. obs.) This has to be related also to the fact that exchanges in horse communities tend to stay internal to that community [10]. Similar trends are observed in the racing industry where not only such neck shapes are considered normal but are considered necessary for good performance (Sénèque et al. in prep.)

## 7. Lack of Knowledge: Abnormal Repetitive Behaviors, Depressed Postures

### 7.1. “False Friends”: Easy Statements about Horses’ Internal State

There are some behaviors that seem to have such an obvious significance that they lead to immediate interpretation about horses’ character or internal state.

Aggressiveness is one of the sources of horses’ culling [90] and is most often considered a temperament trait, especially as it may become persistent [135]. Aggressive horses are just “bad”, “ill-tempered” horses that either have no chance to become appropriate partners or deserve to be “taught” how to behave. In some cases, threats may be attributed to a play behavior, which is still more dangerous as people may not be cautious enough. There is no scientific evidence that aggressiveness has any heritability in horses whereas aggressiveness is part of many pain/sickness scales (e.g., review in [25]) and is known to increase when horses have back problems [117] or are in a poor welfare state, whether because of a lack of feeding resources [136,137], social isolation [138] or overall inappropriate management conditions leading to “bad mood” [139]. Lame Przewalski horses show increased intra-specific aggressiveness [140]. These common false statements are deleterious for horse welfare as they prevent people to question their horses’ health status and to take measures to improve it. 

People do yawn when they are tired, and therefore, yawning in animals is most often assumed to reflect a relaxed state [141]. In horses, this statement has still been reinforced by a line of horsemanship methods, supported by media communication, that indicated that when horses chewed (i.e., vacuum chewing) and yawned, they had understood the orders and were relaxed. In fact, this is forgetting that yawning is a very ambiguous behavior, characteristic of changes in the internal state, but which in animals may reflect pre-sleep situations but also social stress and frustration [142,143]. Studies on yawning in Przewalski horses have revealed that they were more frequent in social groups where aggressiveness was higher [144]. The co-occurrence of high frequencies of stereotypic behaviors and yawning in pre-feeding periods has led Fureix et al. [141] to suggest that high frequencies of yawning may reflect frustration. Frequent observations of vacuum chewing in facilities where welfare conditions are poor suggest also that it may not just be representative of a relaxed state. Yawning/chewing may, in particular, be a mechanism that helps dealing with stress, i.e., going from the sympathetic state fear/flight system and the associated signs such as lack of saliva towards the parasympathetic state (relaxed state), although the whole neurocircuitry involves remains complex to describe (e.g., 143). In any case, it may reflect anxiety and a need for relaxing, that is, the pre-existence of a stressor. It is worth noting that vacuum chewing is considered a stereotypic behavior in sows [145,146]. Whereas chewing and yawning may indeed occur in relaxed states that precede resting, they may as well reflect tenseness and frustration, especially when produced at a high rate and outside a resting context. Being aware of this ambiguous significance would be very important to identify contexts that may be frustrating for horses, both during and outside training.

Play behavior is another critical “false friend”. Play behavior is more frequent in healthy than sick or distressed young animals [147,148,149], which has led quite naturally to the same interpretation in adults. Animals that play look “happy” and “excited”, and given the rarity of clear indicators of positive emotions, this has given rise to a rather consensual view, even in the scientific community, that play, whether adult or young, was a likely candidate for representing good welfare [41,150]. It is common to hear horse owners state that their horse is “happy” because it plays every time it is released in paddock. However, in different species such as horses, play is a purely juvenile feature in natural conditions, where it is almost never observed in adult animals [24,151]. The much higher incidence of adult play behavior in captive or domestic animals is thus particularly intriguing [151]. Recent studies on horses and primates have revealed that adult play behavior was more frequent in facilities where the prevalence of poor welfare was higher [152]. In horses more specifically, adult “players” were presenting a more compromised welfare than non-players (higher oxidative stress, more aggressiveness during tests, more back problems); play was also more frequent in unstable than stable groups [152,153]. Whereas play behavior may trigger short term positive emotions, given its association with opioid release (a common trait with stereotypic behaviors), these data show that it certainly does not constitute a reliable indicator of good welfare. On the contrary, its regular occurrence should rather question whether it may reflect a need for releasing chronic stress and to look at possible remediation. Management conditions will not be changed if horse owners consider their horses as “being happy”. It is also important that protocols proposed to stakeholders for self-assessment of their horses’ welfare state take into account these findings and do not reinforce “folk beliefs” [2].

Because facial expressions are very communicative and reflective of feelings in humans, looking at animals’ faces to assess their internal state seems quite natural [154]. By doing so, attention may be drawn away from more reliable features, such as postural changes, body tensions or simply ears’ positions [119,154,155]. Although the use of facial expressions in pain scales has expanded lately, their reliability in terms of welfare assessment remains to be demonstrated [156]. Moreover, despite detailed descriptions of facial characteristics, their “reading” remains complex which opens up the way to rapid subjective interpretations such as ‘I see that my horse pulls a face”, “smiles”, etc. Over-interpretation of horses’ emotions may easily occur [157]. It seems highly important that validated visible indicators are promoted that may leave less space for personal interpretation. 

### 7.2. Representation of “Good Welfare”

But then how do owners and caretakers consider what horses’ good welfare looks like? Few studies actually have investigated this question. In most studies, the responses concern good practice, that is, resource-based indicators [10]. From a more animal-based point of view, responses can be that horses work or have a good performance which of course is far from being a reliable criterion, but for almost all respondents, welfare implies good health, which does not mean it is easy to recognize (e.g., see sections on gastric or back problems, [6]). Interestingly, for 94% of the respondents in Visser and Van Wijk-Janssen’s study [6], good welfare was represented by horses having “a shiny coat and lively ears “. No mention has been given to calmness nor to calm attention, aspects that may reflect better positive emotions and good welfare than more remarkable excited states such as play sequences [150,158]. 

## 8. Conclusions: Towards Solutions 

Beyond the different “psychological” obstacles mentioned in this review, economical constraints and land restrictions do put limitations to management improvements [3]. Moreover, whereas work, particularly riding, may impair horses’ health and psychological wellbeing, it does not mean horse riding should stop but that it needs re-thinking. 

Promoting horse welfare therefore means finding the right compromises that preserve at best horse welfare but does not impair stakeholders’ welfare (“finding a way of saving the hen and saving the farmer” [34]). Improving horse welfare requires, on one hand innovative integrative proposals that can be easily put into place in order to ensure feasibility and, on the other hand, to accompany stakeholders, but also vets and other reference persons, in developing their ability to decode animal indicators of their internal state. Unfortunately riding schools do not always provide a good example either because they lack themselves of knowledge or through lack of money [4] and comparison of current scientific studies shows that welfare issues may be different in terms of type but not in terms of prevalence between horses managed by professional versus leisure owners. 

Re-thinking practices means working at the system level. Thus, if horses spent more time in free movement and in groups, riding schoolteachers would not need to insist that young riders pull on reins in order to avoid contact with other horses; horses would have less back problems and thus present less undesirable behaviors. Even when land is limited, there are always outdoor or indoor arenas where horses could be released in groups outside work, such as at night time.

Excess weight is less a problem of food quantity than food quality, changing commercial pellets for roughage is not more expensive. Feral horses feed all day long, walk slowly all day long, but do not have much fast gait exercise. Nevertheless, there is no report of overweight horses. Re-thinking pastures and roughage, so as to allow permanent feeding without increasing the prevalence of obesity, is an absolute necessity. Pastures do not need to be “clean”; weeds may be a source of fibers with low energy intake. Preventing overweight horses from feeding may just be counterproductive in that it is a highly frustrating experience which may generate other welfare problems as well as health problems (e.g., gastric ulceration due to empty stomach). Improving feeding strategies may even mean less work and money.

However, in order for stakeholders to find the motivation for such creativeness, there is a need that they “identify” with the horse’s welfare state. Only the transmission of information on reliable, validated and easy to decipher animal-based indicators may help developing the necessary identification, as the closest way to perceiving the animal’s internal state [36]. It is clear that welfare problems are mostly not due to intentional abuse [1], but because of anthropomorphic biases, cultural and personal beliefs, lack of knowledge, and misinterpretations, horse welfare sometimes reflects the saying: “the road to hell is paved with good intentions”. Ensuring that scientific information on animal-based indicators is reliable and transferred to the right networks of transmission has become of primary importance [2]. Identifying and promoting existing good practices is as important as development of “procedural” knowledge may be best promoted by providing examples. Innovative approaches with good results may first surprise and trigger mockery but then lead to active copying. 

## Data Availability

Not applicable.
